# Norwegian adaptation of the Quality in Psychiatric Care – In-Patient instrument: psychometric properties and factor structure

**DOI:** 10.1186/s12913-024-11973-2

**Published:** 2024-12-18

**Authors:** Siri Ødegaard Fossum, Marianne Thorsen Gonzalez, Lars-Olov Lundqvist, Øyfrid Larsen Moen, Agneta Schröder, Hege Skundberg-Kletthagen

**Affiliations:** 1 Faculty of Medicine and Health, Institute of Health Sciences, Norwegian University of Science and Technology (NTNU), P.O box 191, N-2802 Gjøvik, Norway; 2 Faculty of Medicine and Health, University Health Care Research Center, Örebro University, Örebro, Sweden; 3 Faculty of Health and Social Sciences, Department of Nursing and Health Sciences, University of South-Eastern Norway (USN), Drammen, Norway

**Keywords:** Quality of care, Inpatient mental healthcare, Patient perspectives, Instrument, Psychometric properties, Confirmatory factor analysis

## Abstract

**Background:**

Validated instruments measuring the quality of mental healthcare from patients’ perspectives are scarce, and available instruments have been requested. One of the few instruments measuring the quality of care from a patient’s perspective is the Swedish Quality in Psychiatric Care–In–Patient (QPC-IP). This cross-sectional study aimed to translate and adapt the QPC-IP instrument for a Norwegian context and assess its psychometric properties.

**Methods:**

The QPC-IP was translated and adapted to a Norwegian context using a translation back-translation process model. A total of 169 inpatients from specialised mental health services responded to the questionnaire. The QPC-IP comprises six dimensions: *Encounter* (eight items), *Participation* (eight items), *Discharge* (four items), *Support* (four items), *Secluded Environment* (three items), and *Secure Environment* (three items), totalling 30 items. Confirmatory factor analysis was conducted to assess the instrument’s factor structure. Additionally, Cronbach’s alpha was used to establish the instrument's internal consistency.

**Results:**

The results indicated that the Norwegian adaptation of the QPC-IP possesses good psychometric properties, including internal consistency, content, and construct validity, as confirmed by the confirmatory factor analysis results. The confirmatory factor analysis demonstrated an adequate fit for the six-factor structure, consistent with the original Swedish instrument.

**Conclusions:**

The QPC-IP is a user-friendly and easily implementable tool that assesses various dimensions of the quality of inpatient mental healthcare from a patient’s perspective. Moreover, the Norwegian QPC-IP holds potential for use in comparative, cross-cultural studies within mental healthcare services to monitor the quality of the provided services.

## Background

In its initiative for Mental Health 2019 − 2023, the World Health Organisation (WHO) [1] states that everyone should achieve the highest standard of mental health and well-being, indicating that providing good quality mental healthcare is crucial.

Quality of care is a complex issue, and the judgement of quality differs according to the perspectives of politicians, healthcare personnel, and patients, making it challenging to standardise conceptual models of quality of care [2]. Regardless of the perspective on quality of care, there is a consensus that sound instruments for measuring and evaluating quality of care are vital [3]. Moreover, quality in mental healthcare is often best addressed by patient experiences, which must be better understood when aiming to improve quality [4]. Therefore, instruments measuring the quality of mental healthcare should include patients’ perspectives rather than solely those of service providers [5].

The use of validated instruments is crucial [6]. Few validated instruments are available to measure the quality of mental healthcare from a patient’s perspective [7–9]. This makes establishing a baseline from which improved quality can be monitored and evaluated challenging. Moreover, having validated instruments offers opportunities to compare levels of quality in mental healthcare services, both within and between services and countries, which is considered valuable [7, 10]. Hence, to ensure that the available mental health services are of necessary quality, sound and validated instruments are warranted [3].

One of the few instruments measuring the quality of care from a patient’s perspective is the Swedish Quality in Psychiatric Care – In-Patient (QPC-IP), which emphasises aspects important to patients in assessing mental healthcare quality [11]. This instrument has been translated, culturally adapted, and validated in several countries [12–14]. However, the QPC-IP has not been adapted and validated for use in a Norwegian mental healthcare setting, which is the scope of this study.

The quality of mental healthcare from a patient’s perspective is an ambiguous concept; therefore, the operationalisation of this concept with the use of validated instruments needs clarification. Another challenge is that the terms ‘patient satisfaction’ and ‘experienced quality of care’ are often used interchangeably [3], even though ‘quality of care’ appears to represent a more comprehensive concept. Here, ‘patient satisfaction’ has been criticised for being closely linked to subjective expectations of care [3, 15].

A systematic review found 28 instruments assessing patient satisfaction or quality of care in mental healthcare, with 19 of those designed for inpatient care [10]. Common dimensions included overall satisfaction, staff relationships, and skills. Only three instruments measured the quality of mental healthcare, while others focused on satisfaction. The QPC-IP was the sole instrument measuring quality of care from a patient’s perspective [10].

Another systematic review [16] assessing the psychometric properties of 22 instruments measuring the quality of care or satisfaction with care in mental healthcare revealed widely varying psychometric properties; the QPC-IP instrument was reported to have good internal consistency and excellent content validity [16].

The aforementioned systematic reviews revealed that the QPC-IP instrument fulfils the essential requirements to undergo adaptation for assessing the quality of care in Norway. The present paper contributes to further research by focusing on the translation and cultural adaption of the Swedish QPC-IP instrument to the Norwegian context. However, to achieve a valid, reliable, and culturally sensitive measure of a patient’s perspective, psychometric testing is required.

## Methods

### Aim

This study aimed to translate and culturally adapt the Swedish QPC-IP to the Norwegian context and to assess psychometric properties of the Norwegian version of the QPC-IP instrument.

### Design

This psychometric study used a cross-sectional design. It is part of a broader research project using the QPC instruments to address the quality of inpatient mental healthcare in different countries.

### Setting

Norwegian mental healthcare services are organized into specialised health services, such as hospital trusts (Hospitals and District Psychiatric Centres) and community health services (general practitioners, local emergency services, home care, and diverse supported housing contexts) [17]. Four regional health authorities are responsible for providing specialised health services [17]. Generally, these services are publicly funded.

### Recruiting and sample

Patients admitted to mental healthcare wards were the target group for the study and were recruited using a consecutive sampling approach. A stepwise approach was used to recruit participants. Initially, we approached six hospital trusts across the regional health authorities, three of whom agreed to participate. Subsequently, fourteen wards at five locations (Hospitals and District Psychiatric Centres) in the middle and eastern Norwegian regions agreed to participate. Each ward assigned a clinically experienced nurse as the contact person responsible for recruiting eligible participants. The inclusion criteria were that the patients were cognitively capable of completing the questionnaire, had been hospitalised for a minimum of three days before discharge, were ready for discharge, were 18 years or older, and were able to read, understand, and express themselves in Norwegian.

All patients who met these criteria were informed orally and in writing about the study before being formally invited to participate during the last three days of hospitalisation. A total of 169 patients consented to participate and completed the questionnaire.

### Sample size

In planning the study, a sample size of at least 150 participants was calculated based on the COnsensus-based Standards for the selection of health Measurement INstruments (COSMIN) checklist [18], which suggests a minimum of 5 responses per item in the instrument. Therefore, a minimum of 150 participants was required to test the 30-item QPC-IP.

### Data collection

Data were collected through a self-report questionnaire, which was administered between 1st January 2021 and 1st July 2022. Each ward participated for approximately six months. Participants completed the questionnaire during the last three days of hospitalisation and returned the completed questionnaires in a sealed envelope, which was then deposited in a secure mailbox located in the staff area.

Of the 14 wards, 11 delivered information regarding the response rate of the invited participants, which was 61.3%.

Data were collected during the COVID-19 pandemic, and the fluctuating national infection control regulations considerably impacted the data collection process due to the increased workload among healthcare staff.

### The questionnaire and the Swedish Quality in Psychiatric Care – In-Patient (QPC-IP) instrument

The original Swedish 30-item QPC-IP instrument is based on a broad definition of quality of care from a patient’s perspective [19] and has been psychometrically tested among inpatients in Sweden [11]. The QPC instruments are a family of versions for different settings, such as the QPC-OP for mental health outpatient care [20], which has been translated and adapted for community psychiatric care in Norway [21]. The Swedish QPC-IP used in this study has been translated and adapted for Indonesian [13], Spanish [14], and Taiwanese [12] contexts.

The QPC-IP comprises six dimensions: *Encounter* (eight items), *Participation* (eight items), *Discharge* (four items), *Support* (four items), *Secluded Environment* (three items), and *Secure Environment* (three items), totalling 30 items. Table 1 provides excerpts of the items, all of which follow the phrase ‘I experienced that…’. The response options on the four-point Likert-type scale range from “totally disagree’ (1) to ‘totally agree’ (4) for all items. A ‘not applicable’ option is also available [11]. Mean scores above 2.5 were interpreted as high quality [22]. The total Cronbach’s alpha for the original QPC-IP was 0.96, which is considered excellent [11].

**Table 1:** Descriptive statistics and response patterns for QPC-IP dimensions and individual items

QPC-IP **dimensions** and items		Mean	SD	Factor loadings	Max Response (%)	Not applicable (%)	Item missing (%)
	**1. Encounter**	**3.48**	**0.61**				
7	Opportunity to talk when needed	3.41	0.80	0.74	(57.4)	(1.3)	(0)
10	Staff were involved	3.42	0.77	0.77	(57.4)	(0)	(0.6)
11	Staff treated me with warmth and consideration	3.55	0.69	0.82	(65.2)	(0)	(0)
12	Staff cared if I got angry	3.35	0.92	0.77	(43.9)	(26.5)	(0)
15	Staff respected me	3.61	0.78	0.80	(70.3)	(0)	(0)
18	Staff showed they understood my feelings	3.47	0.89	0.81	(61.9)	(1.9)	(0)
20	Staff had time to listen to me	3.50	0.87	0.79	(62.6)	(0.6)	(0)
25	Staff were concerned about my care	3.55	0.75	0.82	(65.2)	(1.3)	(1.9)
	**2. Participation**	**3.11**	**0.68**				
1	I could influence my own care and treatment	3.18	0.84	0.56	(40.6)	(3.9)	(1.3)
5	My opinion of the right care was respected	3.06	0.98	0.81	(38.7)	(5.2)	(1.3)
6	I was involved in decisions about my care	3.01	1.00	0.69	(40.6)	(1.3)	(0)
13	Benefits drawn from earlier experience of treatment	3.27	0.95	0.70	(40.6)	(27.7)	(0.6)
14	I got to recognize signs of deterioration	3.11	0.68	0.55	(38.1)	(14.2)	(0.6)
27	I received information in a way that I could understand	3.22	1.04	0.72	(52.3)	(5.2)	(1.3)
29	I was informed about my mental health problems	3.20	1.00	0.76	(45.8)	(10.3)	(0.6)
30	I received information about treatment alternatives	2.85	1.12	0.72	(32.9)	(15.5)	(0)
	**3. Discharge**	**3.27**	**0.60**				
8	I was offered planning for my continued treatment	3.32	0.85	0.80	(52.3)	(3.9)	(0.6)
16	I was offered a follow-up after discharge	3.62	1.22	0.65	(71.0)	(5.8)	(1.3)
17	I was given help in finding an occupation	2.65	0.79	0.46	(15.5)	(53.5)	(0.6)
21	I knew where to turn after discharge	3.49	0.82	0.69	(63.9)	(7.7)	(0.6)
	**4. Support**	**3.39**	**0.62**				
19	Staff prevented me from hurting others	3.40	0.73	0.53	(32.3)	(46.5)	(1.9)
22	Staff prevented me from hurting myself	3.57	0.99	0.55	(44.5)	(37.4)	(1.3)
23	Nothing shameful about having mental problems	3.33	0.99	0.79	(51.0)	(16.8)	(0.6)
24	Staff said shame must not interfere with seeking treatment	3.26	0.97	0.86	(45.8)	(14.8)	(1.3)
	**5. Secluded environment**	**3.64**	**0.49**				
3	Access to a place that was private	3.79	0.62	0.63	(87.1)	(0)	(0)
26	Had my own room	3.94	0.33	0.63	(96.1)	(0)	(0)
28	Private place where I could receive visits from family	3.18	1.20	0.37	(49.0)	(22.6)	(1.3)
	**6. Secure environment**	**3.40**	**0.62**				
2	Security was high at the ward	3.68	0.65	0.68	(76.1)	(0.6)	(0)
4	Felt secure with fellow patients	3.35	0.86	0.62	(55.5)	(1.9)	(0)
9	Not disturbed by fellow patients	3.18	0.94	0.41	(47.7)	(0.6)	(0)

Dimensions are indicated in bold, while items are in regular font. Mean and Standard Deviation (SD) are provided, along with the number of ’not applicable’ responses and the actual percentage of missing items.The ‘% Max. response’ indicates the proportion of maximum responses. The Likert-type scale ranged from 1(’totally disagree’) to 4 (’totally agree’). All factor loadings were statistically significant (p < 0.001).

Dimensions are indicated in bold, while items are in regular font. Mean and Standard Deviation (SD) are provided, along with the number of 'not applicable' responses and the actual percentage of missing items. The ‘% Max. response’ indicates the proportion of maximum responses. The Likert-type scale ranged from 1 ('totally disagree') to 4 ('totally agree'). All factor loadings were statistically significant (*p* < 0.001).

The self-report questionnaire consisted of two parts: one part with demographic questions and the other with items addressing patients’ experiences of quality of care. Finally, an open-ended question was provided for participants to elaborate on their views on the quality of care.

### Translation of the QPC-IP from Swedish into Norwegian and back translation

The translation process from Swedish to Norwegian followed the steps (1–5) for translation and adaptation of instruments recommended by Sousa and Rojjanasrirat [23]. We used one translator per language considering the project group’s bilingual proficiency, despite two independent translators being recommended [23]. First, the original Swedish QPC-IP instrument [11] was translated into Norwegian by a native-speaking Norwegian and authorszed translator. Second, an independent professional translator with Swedish as their first language, blinded to the original version of the instrument, translated the Norwegian version back into Swedish. This step clarifies the words and sentences used in the translation [23]. Third, the Norwegian (SØF, HS-K, ØLM & MTG) and Swedish (AS & L-OL) project groups compared and discussed the Norwegian translation with the Swedish back-translation to identify any challenges in the translation process and scrutinise the convergence between the translation and back-translation. The two groups made minor changes to obtain the original meaning. This process resulted in the pre-final version of the QPC-IP instrument in Norwegian.

This pre-final version of the QPC-IP instrument was further tested among nine former mental healthcare patients with Norwegian as their first language. They read the questionnaire and evaluated the instructions. Thereafter, they received a form in which they rated whether the items were ‘easy to understand and clear’, ‘acceptable’, or ‘difficult to understand and unclear’. Additionally, they rated the importance of the items, ranking them from one to three (1 = less important, 2 = important, and 3 = very important). After ranking the items individually, the former patients reviewed and discussed them with the researchers (SØF & HS-K) in a cognitive debriefing session, following the recommendations of Sousa and Rojjanasrirat [23]. Overall, the former patients considered the items easy to understand but pointed out that long items should be shortened when possible. They also recognised the questionnaire’s relevance to clinical practice and expressed a desire to have received it during their previous admissions. These considerations were interpreted as good content validity [24]. These former patients added recommendations concerning the readability of the questionnaire design, which resulted in some minor changes aimed at improving face validity [25]. No concerns requiring cultural adaptation were identified during this process.

### Data analysis

Descriptive statistical analyses were performed using SPSS version 28.0.1.0. Confirmatory factor analysis (CFA) was conducted using LISREL 8.80 to assess construct validity, comparing the translated Norwegian version of the QPC-IP with the original Swedish [26]. For the ordinal variables, asymptotic covariance matrices were generated using the PRELIS program [27]. Parameter estimation utilised the weighted least squares method, which is robust with non-normal data [27]. Additionally, to ensure accurate model fit assessment under conditions of non-normality, the Satorra-Bentler scaled chi-square (${\chi }_{SB}^{2}$) test was employed [28, 29].

Based on the QPC instruments manual, questionnaires with 30% or more missing items were discarded before analysis [22]. ‘Not applicable’ answers were considered missing data. After discarding these, the remaining 155 questionnaires were examined for missing data and ‘not applicable’ answers before the CFA analysis. Subsequently, we chose an imputation technique in which the group mean value for each dimension was imputed [30]. This imputation technique was chosen because it aligns with previous studies that have used various versions of QPC instruments [20, 31], facilitating comparability. There were missing or ‘not applicable’ items in 129 questionnaires (Table 1).

CFA assesses construct validity by evaluating the extent to which each item effectively measures the intended dimension and determines whether the items account for the variance in the latent dimensions [29]. Absolute fit indices indicate how the QPC-IP model fits the data and was estimated with the Satorra-Bentler scaled chi-square ${\chi }_{SB}^{2}$, standardised root mean square residual (SRMR), and root mean square error of approximation (RMSEA). The recommended values for these indices are that ${\chi }_{SB}^{2}$ should be nonsignificant with a *p*-value above 0.05, SRMR should be ≤ 0.08, and RMSEA should be ≤ 0.06. Comparative (relative) fit indexes were the comparative fit index (CFI) ≥ 0.95 and the non-normed fit index (NNFI) ≥ 0.95 (also known as the Tucker-Lewis Index [TLI]) [24, 32, 33].

The reliability of the Norwegian version of the QPC-IP was measured by calculating Cronbach’s alpha for the total scale and each of the six dimensions; a value above 0.70 was considered acceptable [24]. In addition, McDonald’s Omega was calculated due to the assumed lack of normal distribution and probable unequal variances, with a value above 0.70 also considered acceptable [34]. However, Cronbach’s alpha was retained to facilitate comparison with other available language versions. A 95% confidence level was used for all the statistical tests.

### Ethical considerations

This study was approved by the Norwegian Regional Ethical Committee (Ref. 113987). The principles of the Helsinki Declaration were followed regarding integrity, confidentiality, and the voluntary nature of participation [35].

The participants received an envelope containing an invitation letter with information about the study, confidentiality procedures, and the procedure for withdrawing their consent. This information was also provided orally. Participants’ informed consent was implied when they completed and submitted the questionnaires in a sealed mailbox located in the staff area. Only the primary investigator had access to this box. The research group could not trace participants' identities. No linking key connected participants to their data, but the questionnaire included a code to track the originating ward. Results were grouped to ensure anonymity, avoiding detailed identification at the hospital or ward level.

## Results

### Characteristics of the study sample

Among the 169 participants who returned the QPC-IP questionnaire, 155 questionnaires were included in the analysis after discarding 14 due to more than 30% missing items. The 155 participants were 65 men and 90 women ranging from 18–93 years old (mean = 40.34, SD = 17.83). Among the participants, 137 had completed high school or college/university education, whereas 29 had completed primary education. Regarding admission status, 113 participants reported being admitted voluntarily, whereas 39 reported being admitted compulsorily (including under compulsory observation). Three participants were unaware of their status upon admission.

### Factor structure of the Norwegian QPC-IP

CFA was conducted on the original Swedish QPC-IP six-factor model, and all factor loadings were found to be statistically significant, as presented in Table 1. The highest factor loadings were observed for the *Encounter* dimension. However, the results of the Satorra-Bentler chi-square test revealed that the model fit was inadequate (${\chi }_{SB}^{2}$ = 451.69; *p* < 0.017) (see Table 2). The other model fit statistics provided satisfactory values, as listed in Table 2.

**Table 2:** Goodness of fit indices of the final model

Index	Current study values	Recommended values
DF	390	
Satorra-Bentler Chi-Square${\chi }_{SB}^{2}$	451.69 (P = 0.017)	Should be non-significant with p-values above 0.05
RMSEA	0.032	≤ 0.06
90% CI RMSEA	0.015; 0.044	
SRMR	0.066	≤ 0.08
CFI	0.99	≥ 0.95
NNFI (TLI)	0.99	≥ 0.95

The Cronbach’s alpha of the Norwegian QPC-IP was found to be good overall (α = 0.94), but not for all the individual dimensions that varied from 0.40 to 0.93, as shown in comparison with research on other QPC-IP language versions in Table 3. Additionally, McDonald’s Omega values for the Norwegian QPC-IP are also presented.

**Table 3:** Cronbach’s alpha of QPC-IP in the current and previous studies. (McDonald’s Omega for the current study in parentheses)

**Dimensions**	***N* of items**	**Current study** **(*N*** **= 155)** Norway		**Schröder et al. (2010)** Sweden	**Lundquist et al. (2018)** Indonesia	**Sanchez-Balcells et al. (2021)** Spain
Total Scale	30	0.94	(0.95)	0.96	0.70	0.94
Encounter	6	0.93	(0.93)	0.95	0.54	0.89
Participation	6	0.88	(0.88)	0.91	0.42	0.85
Discharge	4	0.75	(0.76)	0.80	0.28	0.71
Support	4	0.78	(0.77)	0.89	0.36	0.89
Secluded environment	3	0.40	(0.41)	0.77	Not estimated^a^	0.68
Secure environment	3	0.61	(0.64)	0.75	0.46	0.74

^a^The secluded environment dimension did not fit the model and was excluded from the final Indonesian version

### Descriptions of quality of inpatient care

**Fig. 1:**
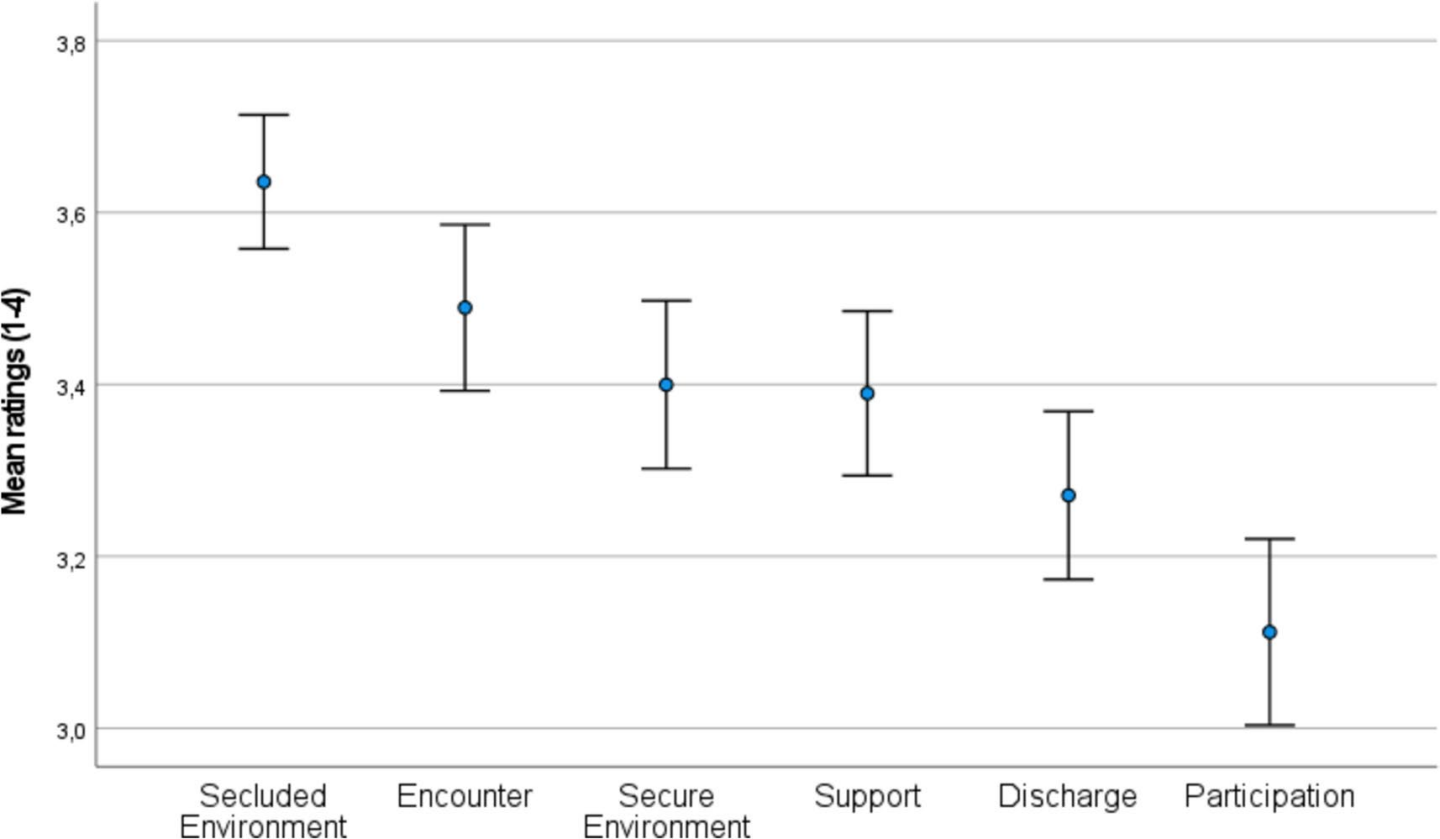
Mean ratings of the Norwegian version of the QPC-IP dimensions. Error bars represent a 95% Confidence interval

Table 1 presents the means and standard deviations of each item of the QPC-IP questionnaire, along with the percentage of maximum scores for each item. Based on the results of *t-*tests and the ranked dimensions, as shown in Fig. 1, participants rated a significantly higher perceived quality in the *Secluded Environment* dimension compared to the second-ranked dimension *Encounter* (*t*_(154)_ = 3.023, *p* < 0.003). However, there were no significant differences between *Encounter* and *Secure Environment* (*t*_*(154)*_ = 1.882, *p* < 0.062) or between *Secure Environment* and *Support* (*t*_*(154)*_ = 0.197, *p* < 0.844). Conversely, *Support* was perceived as significantly higher than *Discharge* (*t*_*(154)*_ = 2.469, *p* < 0.015), which was perceived as significantly higher than *Participation* (*t*_*(154)*_ = 3.754, *p* < 0.001). Additionally, *Encounter* was perceived as significantly higher than *Support* (*t*_*(154)*_ = 2.829, *p* < 0.005).

The high percentages of maximum scores on the individual items indicated a ceiling effect in the distribution of data.

## Discussion

This study aimed to translate and culturally adapt the Swedish version of the QPC-IP for the Norwegian context to assess the psychometric properties of the Norwegian QPC-IP instrument. The CFA analysis demonstrated an adequate fit of the six-factor structure, consistent with the original Swedish version. While the goodness of fit indices were adequate, the ${\chi }_{SB}^{2}$ test was significant.

### Issues related to the analysis of psychometric properties

Cronbach’s alpha for the total Norwegian QPC-IP instrument indicated good reliability. In other words, the different items appear to measure the same phenomenon—the quality of mental healthcare. McDonald’s Omega showed slightly higher values than Cronbach’s alpha but did not alter the overall internal consistency estimate for the total scale or the individual dimensions. Cronbach’s Alpha will be discussed due to its relevance for comparison and because it provides a more conservative estimate [34].

Table 3 shows that Cronbach’s alpha results in the current study are similar to those in the Spanish adaptation [14] but showed slightly lower values than the original Swedish version [11]. Notably, the Indonesian version had a lower Cronbach’s alpha than the other translated versions [13], which may be explained by cultural and organisational differences.

Considering the results on the individual dimensions *Secluded Environment* and *Secure Environment*, the Cronbach’s alpha was not satisfactory, which was inconsistent with the Cronbach’s alpha reported in the original Swedish version [11]. However, these dimensions were also the weakest in the original Swedish version [11]. Additionally, several other studies reported a low Cronbach’s alpha for the *Secluded Environment* dimension [12–14]. Furthermore, owing to nonsignificant or negative loadings at the item level, the *Secluded Environment* dimension was excluded from the final Indonesian version [13]. Regarding the *Secure Environment* dimension, all language versions except for the Indonesian version reported satisfactory results for Cronbach’s alpha [12, 14]. The fact that the dimensions *Secluded Environment* and *Secure Environment* have three-item scales might somewhat explain the low Cronbach’s alpha values, as scales with more items typically achieve higher Cronbach’s alpha values [24].

Surprisingly, the *Secluded Environment* dimension in the Norwegian version had the lowest Cronbach’s alpha among all the language versions, an issue that should be further explored. Particularly, it would be interesting to compare this with the results of the Swedish version [11] as Norway and Sweden have similar languages, healthcare systems, and cultures. These deviating results in the Norwegian version may be explained by the data being collected during the COVID-19 pandemic. Some wards reported obvious deviations from their normal practices during this period as national regulations prevented wards from allowing visitors. Moreover, not receiving visitors might have affected the rates for item 28, addressing whether the patients were allowed to receive visitors in a private location.

This study assessed construct validity using CFA, which yielded a six-factor model (six dimensions) consistent with that of the original QPC-IP [11]. Moreover, all item loadings exceeded the acceptable minimum of 0.30 [36], indicating a moderate to strong relationship with the underlying construct. The goodness-of-fit indices supported a good model fit [24], except for the Satorra-Bentler scaled chi-square test results (Table 2). According to Polit and Yang [24], the chi-square test is sample size sensitive; and when other fit statistics indicate a good fit, it may be reasonable to conclude that the model is adequate. Therefore, the CFA results provide evidence for the satisfactory construct validity of the Norwegian version of the QPC-IP. These results align with previous studies using QPC-IP [11–14].

### Issues related to the quality of mental healthcare 

The participants rated the highest quality in the *Secluded Environment* dimension, thus contradicting the consistent results from previous studies that used the QPC-IP instrument, where the *Encounter* quality dimension was rated highest [11–14]. The *Encounter* dimension was also rated highest among patients and relatives in the Norwegian version of the QPC for community outpatients (QPC-COP) and outpatients’ next of kin [21] as well as in the Norwegian QPC-COPS instrument assessing mental healthcare staff perceptions [37, 38]. However, our results are supported by a national Norwegian survey addressing mental healthcare inpatients, which reported that they were most satisfied with issues related to the environment’s structure and facilities [39]. Nevertheless, direct comparisons cannot be made even though the scales in Bjertnaes and Iversen’s [39] study share similarities with the QPC-IP instrument. Thus, standardised and validated instruments for assessing quality of care are crucial in facilitating benchmarking and comparisons across mental healthcare services worldwide [7, 10]. Using such instruments, researchers and healthcare providers can evaluate and improve quality of care in a meaningful and systematic manner, leading to improved patient outcomes and experiences.

The participants rated the *Participation* dimension as having the lowest quality, which concurs with previous studies using the QPC-IP [11, 13]. Despite Norwegian national legislation and regulations emphasising patient involvement in clinical decisions [17, 40], there may still be room for improvement on these issues. Several possible obstacles to patient involvement have been identified in previous research, such as symptom pressure, lack of insight, inadequate information provision, medical jargon, and a lack of a clear definition of participation as well as experience in participation [41–43]. However, the current study did not investigate specific strategies and interventions implemented in the wards for promoting patient participation. Patient involvement is associated with securing empowerment and satisfaction with care, which suggests the importance of participation in enhancing patient outcomes [43]. It was anticipated that increased patient participation would also increase the perceived quality of care.

According to the results, certain individual items in the Norwegian version of the QPC-IP may not be relevant or applicable to all study participants. For example, more than half of the participants rated item 17, ‘I was given help finding an occupation’, as ‘not applicable’ (Table 1). This result may reflect a lack of content equivalence [24] owing to differences in healthcare services between Norway and Sweden, as assistance with finding an occupation is typically provided by the Norwegian Labour and Welfare Administration rather than within the specialist healthcare services. Considering these results, future research employing the QPC-IP in Norway may benefit from reconsidering the inclusion of item 17 or modifying it to better align with the cultural context of the Norwegian healthcare system.

### Limitations and strengths

This study’s small sample size is a potential limitation, but determining an appropriate sample size for a CFA is a complex process that depends on various factors, such as model size and complexity, variable distribution, missing data, variable reliability, and relationship strength between the variables [44]. Although some guidelines suggest a sample size of 5–10 participants per item, others recommend at least 200 participants [24]. The sample size aligns with the minimum recommendation from COSMIN [18] and is consistent with similar studies on QPC-IP that utilised comparable sample sizes and reported consistent CFA results [11, 13, 14]. However, due to the small sample size, the results of this study may not be generalisable to the broader population.

The selected imputation technique for missing data has been criticised for artificially narrowing confidence intervals and reducing data variability [45]. This could bias the interpretation of results, particularly for items or dimensions with a high percentage of “not applicable” responses. This limitation arises because the imputation technique may not accurately reflect the true variation in the missing data, potentially compromising the accuracy of analysis and the conclusions drawn from it [45].

Furthermore, a high proportion of participants rating quality at the maximum level on several individual items indicates a ceiling effect, prompting questions about the instrument’s sensitivity to detect variations in quality of care [46]. Addressing this challenge in future research could involve extending the response scale [46].

Despite these limitations, a major contribution of this study is that it addresses patients’ availing mental healthcare—a population whose opinions are vital regarding their experience of the quality of services they receive. Moreover, translating, culturally adapting, and psychometrically testing a recognised instrument measuring the quality of care for this group is considered a strength. Additionally, the high response rate of 61.3% in this sample is considered a strength, indicating active participation. This study also provides valuable insights into participants’ assessments of the experienced quality of the received services.

## Conclusions

Despite the study’s limitations, the Norwegian QPC-IP demonstrates sufficient psychometric properties, including internal consistency and construct validity, as confirmed by the CFA results. The CFA indicated an adequate fit for the six-factor structure, consistent with the original Swedish version. The QPC-IP is a user-friendly tool that effectively assesses various dimensions of inpatient mental healthcare quality from a patient’s perspective.

### Implications for future research

Future research should employ a test–retest method to further assess the robustness and enhance the reliability and applicability of the QPC-IP in the Norwegian context. Researchers should also consider revising items that received numerous “not applicable” responses and extending the response scale to prevent ceiling effects. Comparative research between healthcare services in Norway and Sweden could identify factors contributing to the instrument’s limitations in Norway, thereby improving its relevance and validity across different contexts. Testing for additional aspects of measurement invariance, such as metric and scalar invariance, could further validate the instrument. Additionally, exploring the possibilities for identifying an instrument suitable for examining discriminant validity would be beneficial. The QPC-IP holds potential for use in comparative, cross-cultural studies within mental healthcare services to monitor service quality. Combining feedback from healthcare staff using the QPC-Inpatient Staff instrument [47] and from patients using the QPC-IP could also be valuable for continuous quality improvement efforts within healthcare services.

## Data Availability

The datasets generated during this study are not publicly available, as a subsequent paper will be prepared based on this data. However, they can be accessed from the corresponding author upon reasonable request.

## Abbreviations

CFA: Confirmatory Factor Analysis
CFI: Comparative Fit Index
DF: Degrees of Freedom
NNFI: Non-Normed Fit Index
RMSEA: Root Mean Square Error of Approximation
SRMR: Standardised Root Mean Residual
TLI: Tucker-Lewis Index
QPC-IP: Quality in Psychiatric Care – In-Patient instrument
${\chi }_{SB}^{2}$: Satorra-Bentler Chi-Square
